# Predicting Box-Office Markets with Machine Learning Methods

**DOI:** 10.3390/e24050711

**Published:** 2022-05-16

**Authors:** Dawei Li, Zhi-Ping Liu

**Affiliations:** 1 School of History and Culture, Shandong University, Jinan 250100, China; lidawei@sdu.edu.cn; 2 School of Control Science and Engineering, Shandong University, Jinan 250061, China

**Keywords:** box-office prediction, economic systems, time-series data, support vector machine, machine learning

## Abstract

The accurate prediction of gross box-office markets is of great benefit for investment and management in the movie industry. In this work, we propose a machine learning-based method for predicting the movie box-office revenue of a country based on the empirical comparisons of eight methods with diverse combinations of economic factors. Specifically, we achieved a prediction performance of the relative root mean squared error of 0.056 in the US and of 0.183 in China for the two case studies of movie markets in time-series forecasting experiments from 2013 to 2016. We concluded that the support-vector-machine-based method using gross domestic product reached the best prediction performance and satisfies the easily available information of economic factors. The computational experiments and comparison studies provided evidence for the effectiveness and advantages of our proposed prediction strategy. In the validation process of the predicted total box-office markets in 2017, the error rates were 0.044 in the US and 0.066 in China. In the consecutive predictions of nationwide box-office markets in 2018 and 2019, the mean relative absolute percentage errors achieved were 0.041 and 0.035 in the US and China, respectively. The precise predictions, both in the training and validation data, demonstrate the efficiency and versatility of our proposed method.

## 1. Introduction

The culture industry is one of the most important and influential parts of the world economy [1]. Movies are a popular relaxation method for young and diverse-aged people in all individual countries. The development status of the movie industry specifically reflects the consumption patterns and economic growth of a country [2]. During the modernization processes, the movie industry is expected to play increasingly crucial roles in the capital and commerce of cultural economics due to its popularity. The total box-office revenue of a country is a valuable index of investment and finance for decision-makers, such as producers and moviemakers. In this sense, early prediction of box-office incomes is very beneficial for movie market-related capitalization and data-driven administration.

Accurate prediction of movie box-offices is still significant and challenging economically due to its extreme dynamics. The complexity is highly related to the entropy of the underlying cultural-economic system [3,4], especially in a time-series study over a temporal range [5]. Due to its importance, Litman proposed a prediction study for the financial success of theatrical movies as early as 1983 [6]. He focused on various aspects of the movie industry, such as the marketing and distribution pattern and cinema arrangement. From the perspective of prediction methods, some studies so far have been proposed to meet this challenge [7], although most of them have not reached satisfying prediction performances and applicable practices [8,9]. The econometric methods, e.g., linear regression and log-linear regression, have been introduced to box-office prediction. Elberse and Eliashberg proposed such a method for a motion picture in domestic markets and in foreign markets by examining the interrelationship between the different determinants of box-office revenue [10]. The machine learning-based methods, such as neural networks, have been employed to forecast the box-office revenue of a movie before its theatrical release [11]. Ahmed et al. built up such a prediction system using hybrid methods for box-office success quotient forecasting [12]. Recently, some studies have been proposed for the instant prediction of the success of a movie at the box-office based on the big data of social media [13], Google search [14] and Wikipedia page activity [15]. However, these available methods focus only on the box-office success of one movie. There are still few studies for predicting the gross movie market of a country [16]. Moreover, the predictor variables in these methods consider few effects of the economic factors. Box-office markets have very complicated relationships with many factors, ranging from macroeconomics of gross domestic product, per capita income, number of theater screen and online-storytelling media to the director, screenwriter, costars, moviegoers and consumption traditions [17,18]. Effective predictions need to be conducted using reasonable and easily-accessible predictor variable(s). Furthermore, the prediction method needs to consider the time-course data of box-offices rationally and thoroughly. The effective and efficient prediction of gross box-office revenues in one country will provide a deep understanding and valuable direction of investments in the economy and management of the culture industry.

To this end, we aim to propose a machine learning method for predicting a nationwide box-office market through its associated economic factors. To select the prediction algorithm, we investigated various machine learning-based methods, e.g., support vector machines, random forest and neural networks and econometrics-based models (e.g., linear, ridge and auto regressions), to acquire the relationship between these economic indices (predictor variables) and the box-office (response variable). When selecting the prediction variables, we encoded the box-office markets using various predictor variables, e.g., gross domestic product (GDP) and the number of movie screens (NMS) and identified the most reliable and easily-available indices for practical predictions. For proof of concept, we empirically tested our prediction performances using a sliding-window encoding strategy for these time-series studies of box-offices in the US and China. We compared multiple methods with alternative dependent variables to identify the most efficient prediction strategy. We concluded that the gross box-office markets are predicable using a support vector machine with GDP in the case study of two countries. Furthermore, the accurate predictions over the past several years from 2017 to 2019, prior to the COVID-19 pandemic, provide more evidence for the effectiveness and advantage of our proposed prediction method.

For clarification, the contributions and novelties of this paper are summarized as follows:

- We propose an SVM-based method to predict the global box-office market of a country by its economic factor of GDP.
- We implemented four machine learning methods and four econometric methods with diverse combinations of economic factors as prediction variables. The comparison results in both the US and China box-office markets highlight the selected prediction strategy according to prediction performances.
- The time-series cross-validation and the mimicked prediction of the box-office market in real application scenarios prove the effectiveness and efficiency of our proposed method of predicting nationwide box-offices. The easy availability of economic factors also implies its flexibility.
- The empirical experiments with different combinations of economic factors indicate their diverse effects on box-office prediction. The selected prediction variable of GDP proves its interpretable close relationship with box-office revenues.

## 2. Materials and Methods

### 2.1. Data

To implement our prediction methods, we collected the statistical data of the two largest movie markets, i.e., the US and China, from a variety of data sources. Due to a reform policy released in 2001 in China after joining the WTO (World Trade Organization), the operational management of movies and their related release policies are significantly different from the previous era. Thus, we collected data from the box-office markets in these two counties from 2002 onwards. In the prediction models, the box-office refers to the outcome or response variable. For easy accessibility, we collected GDP and NMS in each country as the predictor or dependent variable. In detail, the box-office and the NMS data of the US were collected from NATO (National Association of Theatre Owners) and the GDP data of the US were obtained from the IMF (International Monetary Fund). The corresponding values of China were obtained from the National Bureau of Statistics of China and was adjusted to the same units and scale of the data from the US.

It is worth noting that the COVID-19 pandemic is currently threatening all human health. Most countries are still implementing social distancing and movie theaters are still shut down. That is to say, movie box-offices as well as current economic factors are not in their normal states. We began this project before the pandemic. Thus, we performed our study on the data collected before the ongoing pandemic caused by the SARS-CoV-2 virus. The proposed strategy will be still valid when the world economy and consumption recovers from the pandemic.

**Figure 1:**
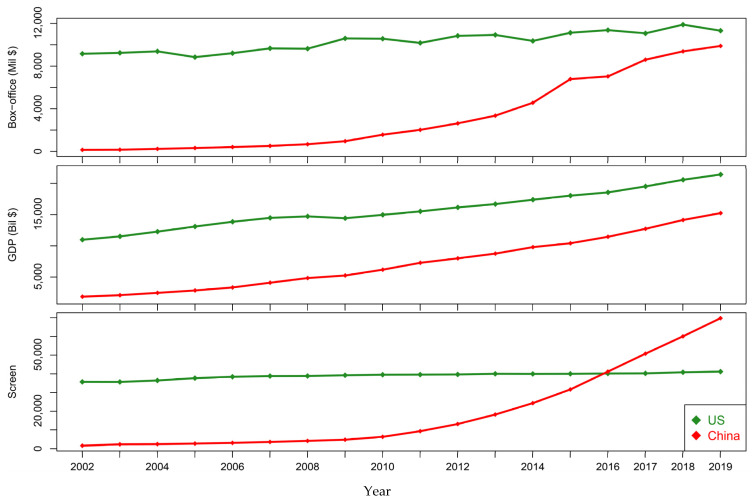
The box-offices over the period 2002 to 2019 in US and China. The corresponding GDP and NMS are also illustrated simultaneously.

Figure 1 records the gross movie box-office markets from 2002 to 2019 in the US and China. In total, we collected 18 continuous time-series data of movie box-offices, GDP, and NMS for the two countries. The task here is to predict the time-series of box-offices using these economic factors. We used the sequential data from 2002 to 2016 for the training and selection the prediction methods and used the data from 2017 to 2019 for testing and validation purposes.

### 2.2. Framework of Prediction

**Figure 2:**
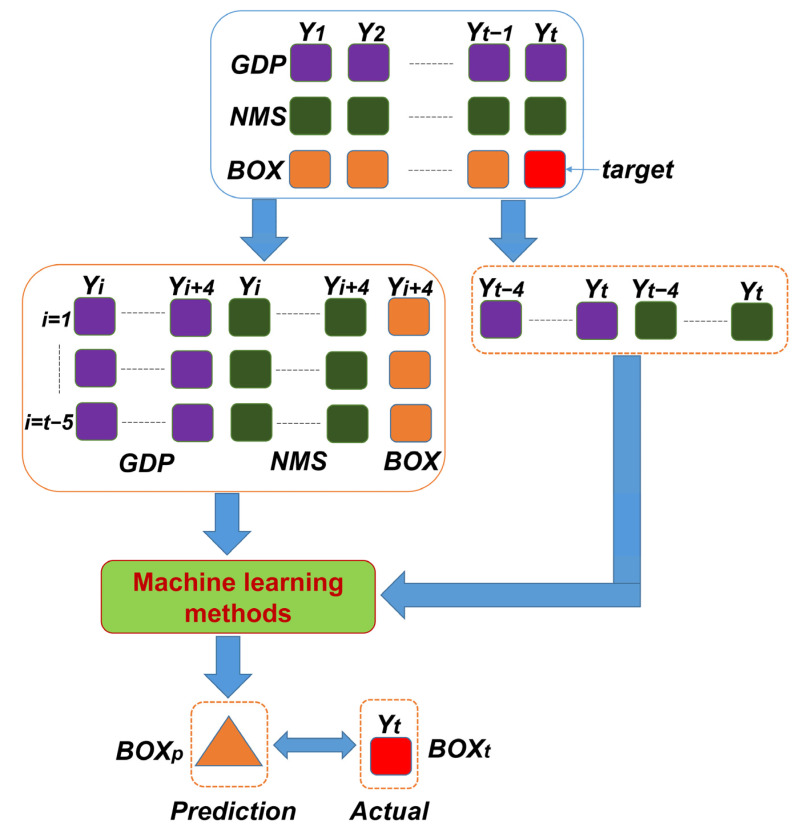
The framework of predicting box-office markets by machine learning methods.

Figure 2 demonstrates the framework of predicting the gross box-office revenues with machine learning methods. Firstly, we used the available global economic index and the local number of resources as the prediction factors (i.e., the independent variables of GDP and NMS) and the box-office in the *t* year as the value (i.e., dependent variable BOX) that we aimed to predict, i.e., the target, as shown in Figure 2. We collected the data of GDP, NMS and BOX from 1 to *t* year. Secondly, we reorganized the data by combining the predictor indices, GDP and NMS, in the year of *Y*_1_, *Y*_2_, *…*, *Y_t_*_−1_ as the training datasets. The corresponding *GDP_t_* and *NMS_t_* and the true box-office of *BOX_t_* in the year of *T_t_* was used as the testing data for evaluating the prediction performances. By setting up a sliding-window of *S* years (set *S* = 5 via empirical experiments), we combined the corresponding dependent variables (GDP and NMS) and the response variable (BOX) into a training pair. That is to say, we regarded the *BOX_i+_*_4_ of the year *Y_i+_*_4_ to be a response of these values of GDP and NMS in the last 5 years from *Y_i_* to *Y_i+_*_4_. The values of GDP and NMS as feature variables are encoded into a vector representing the factors.

After we input the *t* − 5 pairs of training data into a machine learning method and achieved the appropriate relationality between these dependent variables and the response variables, we were able use these trained machine learning methods to forecast the box-office of the year *t*. Then, the box-office outcome of the year *t* was predicted by the last 5 years’ *Y_t_* to *Y_t_*_−4_ of GDP and NMS. The difference between the actual box-office *BOX_t_* and the prediction *BOX_p_* was evaluated to assess the effectiveness and efficiency of the trained machine learning method.

As for machine learning techniques, we collected and tested some popular methods, such as support vector machine, random forest and neural network, to compare and select the most suitable method for prediction. There were also some baselines of predicting time-series data by traditional econometric methods, such as linear regression and ARIMA (autoregressive integrated moving average). For comparison, we also implemented these conventional methods in statistics in order to check their abilities of the box-office prediction. In total, we implemented eight methods for our empirical experiments on the data from the US and China. We also tested the combination (‘GDP + NMS’) and separation (‘GDP’ or ‘NMS’) of the predictor variables in the feature encoding procedure to seek suitable dependent variables and combinations.

### 2.3. Machine Learning Methods

#### 2.3.1. Support Vector Machine

Generally, box-office prediction can be viewed as the task of first finding a function Y=f(X) between the response variable (box-office) and dependent variables (GDP and/or NMS) from the training data. After the function is determined, the prediction can be easily achieved after inputting new economic factors. Originally, support vector machine (SVM) was widely used in classification [19]. It aims to seek two hyperplanes of supporting vectors with maximal margins distinguishing two types of data points. Alternatively, we employed the technique of regression analysis [20]. The same idea of identifying support vectors is crucial in the SVM-based regression analysis. Suppose the function takes a linear form as $Y=f(X)=\sum_{i=1}^{t}W_{i}X_{i}+b$, then the coefficients can be identified by solving a quadratic programming, i.e.,

(1) $$\begin{array}{cc} \min\;\frac{1}{2}{\left\|W\right\|}^{2}+c & \sum_{i=1}^{t}(\xi_{i}+\xi_{i}^{*})\\ s.t.\;Y_{i}-WX_{i}-b & \leq \varepsilon +\xi_{i},\\ WX_{i}+b-Y_{i} & \leq \varepsilon +\xi_{i}{}^{*},\\ \;\;\;\;\xi_{i},\xi_{i}{}^{*}\geq 0, \end{array}$$

where $\xi_{i}$, $\xi_{i}^{*}$ are introduced as slack variables in the case of feasibility and c is a trade-off coefficient [19,20]. For nonlinear cases, the learning algorithm employs kernel tricks, e.g., a Guassian radial basis function kernel, to transform the data into a feature space with higher dimensions. In the mapped data space, it is possible to achieve better regression and prediction thereafter. We implemented the SVM regression based on the ‘e1071′ package in R. The parameters such as ‘kernel’ and ‘gamma’ were optimized empirically, namely, we ran the options and chose the one with relatively better prediction performance metrics (see Equations (6) and (7) below).

#### 2.3.2. Random Forest

Random forests (RF) is an ensemble machine learning method for generating numerous decision trees by randomly selecting features [21]. It applies the bagging techniques of sampling sets to train decision-tree learners. For individual decision trees, RF defines a margin function to measure the probability of the votes at (*X*, *Y*) for the right class to exceed the vote for any other class, i.e.,

(2) $$mf(X,Y)=\frac{1}{t}\sum_{i=1}^{t}\left\{I(h(X)=Y)-\underset{j\neq Y}{\max}I(h(X)=j)\right\},$$

where I(·) refers to the indicator function. Based on *mf*, two parameters, ‘strength’ and ‘correlation’, of a random forest are defined and optimized for classification. In regression, each decision tree changes to take on numerical values instead of class labels [21]. Then the prediction is made by averaging the predictions of all the individual trees. It is proven to avoid overfitting in training and to achieve good generalization ability in prediction [22]. Here, RF was implemented via the ‘randomForest’ package in R. In detail, the regression mode is assumed. For simplicity, we kept the default argument values in the calling function because there was only a slight difference between tuning parameters. In detail, the impurity measurement metric was the Gini index in the 500 decision trees.

#### 2.3.3. Neural Network and Deep Neural Network

Neural network (NN) simulates the information processing of neurons in the brain [23]. They model a complicated collection of connected nodes (artificial neurons), where information transmits from one node to another. The neurons receive the signals from others and transfer them to the other connected neurons. The neuron takes the inputs of the predictor variables of GDP and/or NMS and outputs the prediction of the box-office, i.e.,

(3) $$Y_{t}=f(W^{T}X)=f(\sum_{i=1}^{t}W_{i}X_{i}+b),$$

where f(·) is an activation function, such as a sigmoid function, and W denotes the weight parameters associated with the connections between neurons. The weight between neuron nodes are typically trained with some specific version of the back-propagation (BP) algorithm [24]. Deep neural network (DNN) is an improved version of the artificial neural network with multiple hidden layers. An example class is convolutional neural network in which the multilayer perceptions are achieved with minimal preprocessing of original data [25]. We implemented the two types of neural network, i.e., NN trained by the BP algorithm and DNN with the initialized weights using a deep belief network, to predict movie box-offices. By employing the ‘nnet’ package in R, we tested various neuron numbers, i.e., 3, 5, 7 and 9, in the hidden layer of NN architecture and choose the one with 5 neurons based on its relatively better prediction performance. Similarly, we empirically determined the vector of ‘(5, 3)’, containing the number of hidden nodes in each hidden layer during the call of the ‘dbn.dnn.train’ function in the ‘deepnet’ package.

### 2.4. Econometric Methods

#### 2.4.1. Linear Regression, Log-Linear Regression and Ridge Regression

Linear regression (LR) is a fundamental statistical model used to describe the linear relationship between response variables and dependent variables [26], i.e.,

(4) $$B_{t+1}=\sum_{i=1}^{t}\alpha_{i}X_{i}+\varepsilon_{t+1}.$$

where $B_{t+1}$ is the box-office in the year of t+1, $\alpha_{i}(i=1,\ldots ,t)$ is the coefficient of the dependent variable, $X_{i}$ was used for the prediction and $\varepsilon_{t+1}$ is the error term. The assumption of the linear combination of the weighted coefficients in these variables provided the direct explanation of the predictor through these measurable indices. After these coefficients were determined by the available data, the future box-offices were easily predicted by the fitted equation.

To remove the scale impact of these variables, the logarithm function, i.e., log-linear regression (LLR), can be employed to transform the response and dependent variables. In the transformed space, the regression achieves possible linear relationships [26]. To mitigate the problem of multicollinearity in LR, we also employed ridge regression (RR) to achieve the parameter estimation with a tolerable amount of bias [27]. Specifically, a *L*_2_-norm penalty term of parameters, i.e., $\sum_{i=1}^{t}\alpha_{i}^{2}$, was introduced to the loss function, such as the mean squared error in the parameter estimation. The regularized term was also expected to prevent the linear regression model from overfitting by shrinking some parameters to a smaller size [28]. For convenience, we use the basic functions in R to perform the LR and LLR predictions and the ‘lm.ridge’ function in ‘MASS’ package to implement the RR model.

#### 2.4.2. ARIMA

ARIMA is often used to fit the time-course data for prediction in the series [29]. In ARIMA, the original time-series data is transformed by differencing to make it stationary. Mathematically, let *d* be the order of differencing:

If d=0, ${\tilde{X}}_{t}=X_{t}$.

If d=1, ${\tilde{X}}_{t}=X_{t}-X_{t-1}$.

If d=2, ${\tilde{X}}_{t}=(X_{t}-X_{t-1})-(X_{t-1}-X_{t-2})=X_{t}-2X_{t-1}+X_{t-2}$.

The ARMIA model is

(5) $${\tilde{X}}_{t}=\varphi_{1}{\tilde{X}}_{t-1}+\cdots +\varphi_{p}{\tilde{X}}_{t-p}+\varepsilon_{t}-\theta_{1}\varepsilon_{t-1}-\cdots -\theta_{q}\varepsilon_{t-q},$$

where t∈Z is the time point. The first part is the AR part, *p* is the order of time lags of the autoregressive model, *q* is the order of the moving-average model. We employed the automatic ARIMA to determine the best parameters of (*p*,*d*,*q*) in our predictions [29]. Specifically, the ‘auto.arima’ function in the ‘forecast’ package conducts the best ARIMA model search over other possible models, according to some information criteria (e.g., Akaike’s information criterion). We use the default parameters in the calling function for its embedded search strategy.

### 2.5. Prediction Performance Evaluation

Sequentially, we implemented the predictions in the time series of the box-office. For the predicted box-offices and the actual data of these individual testing years, we employed the relative absolute percentage error (*RAPE*) to evaluate the prediction performance, i.e.,

(6) $$RAPE=\frac{\left|{\hat{B}}_{t}-B_{t}\right|}{\left|B_{t}\right|},$$

where ${\hat{B}}_{t}$ is the prediction for the year *t* and $B_{t}$ is the actual box-office. For evaluating the prediction error in all testing years, the following relative root mean squared error (*RMSE*) is proposed as the metric to evaluate the prediction performance, i.e.,

(7) $$RMSE=\sqrt{\frac{1}{n}{\sum_{i=1}^{n}\left(\frac{{\hat{B}}_{i}-B_{i}}{B_{i}}\right)}^{2}}.$$

## 3. Results and Discussion

We performed empirical experiments to predict box-office markets in the US and China. We first implemented eight methods individually for the comparison and evaluation of these prediction methods. To select the predictor variables of GDP and NMS, we tested their prediction performances through individual (independent) and integrated (combination) processes, with the possible alternatives of introducing the past values of the box-office. When we identified the efficient method, i.e., SVM, and the easily-available predictor variable, i.e., GDP, in the prediction, we validated the performance with the actual box-office markets in 2017. The prediction results for the two consecutive years of 2018 and 2019 were also presented by mimicking future real application scenarios.

### 3.1. Predictions by Different Methods

To select a suitable prediction method, we compared the four machine learning methods and the four econometric methods in the sequential prediction of the time-series of the box-office from 2012 to 2016. Essentially, the prediction is to correlate the GDP and/or NMS with the box-office market. Table 1 lists the prediction performances by the eight prediction methods with the predictor variables of GDP and/or NMS.

In detail, the SVM-based prediction method achieves a mean RAPE of 0.002 for the US and of 0.011 for China in 2016 and a RMSE of 0.056 for the US and of 0.183 for China over the testing period of five years, from 2012 to 2016. The predictor variable NMS seems to achieve better predictions than GDP. The performance using their combination is located in the middle. These results can also be observed using other prediction methods, such as RF, NN and DNN, while the econometric predictions based on LR by GDP are better than those by NMS.

Moreover, the predictions of these machine learning methods for the US are often better than those for China. While the predictions of the econometric models for China are often better than those for the US. For instance, in the predictions of the US with the predictor variable GDP, the RMSE of RF achieves 0.041 in the five years. In China, it achieves a high RMSE of 0.552. When we employ the LR method, it achieves the RMSE of 0.292 in China and of 0.574 in the US. This implies the ineffectiveness of these traditional statistical methods in the prediction of nationwide box-office markets. LLR slightly improves the prediction performances because the logarithm transformation of the data enhances the linear relationship between the response variable and dependent variables. Compared to LR and LLR, RR achieves better performances because we removed the collinearity of the slide-windowing encoded features in this method. In ARIMA, the predictions had smaller RMSE values for China than those for the US.

The different performances in different methods imply the suitability of each method in each case study. Additionally, they indicate the diverse patterns of the box-office markets in the US and China. The box-offices in US are often very stable due to the country’s relatively mature economics. Due to the rapid economic and social development in China, its movie market is very dynamic with a fast up-growth tendency. The predictor variables in the US and China also correspond to these indications. From the prediction performances of these econometric methods, there are no clear (log-) linear relationships between the box-office and GDP and/or NMS. The machine learning methods can identify the latent nonlinear relationships between them and thus result in relatively better predictions. 

In Table 1, for each method, we highlight the best predictions with the lowest RMSE values by bold fonts in the US and China respectively. Please note that we also selected the most suitable parameters in these machine learning methods. For instances, when we tested the prediction by SVM, we tested the different RMSE values with different kernel functions, such as radial and sigmoid. In the predictions by NN and DNN, we also optimized the parameters by empirical tests, e.g., node number of hidden layers and number of hidden layers. The performances listed in Table 1 are the relatively better ones in these tests. As shown, in the time-series cross-validations, the predictors were all sensitive to the changes of different inputs. In the regularized model of RR, the RAPE and RMSE are also diverse in the sequential year predictions. Moreover, the combinations of different variables obviously affected the prediction performances. Thus, there are no overfitting issues in our prediction models.

**Figure 3:**
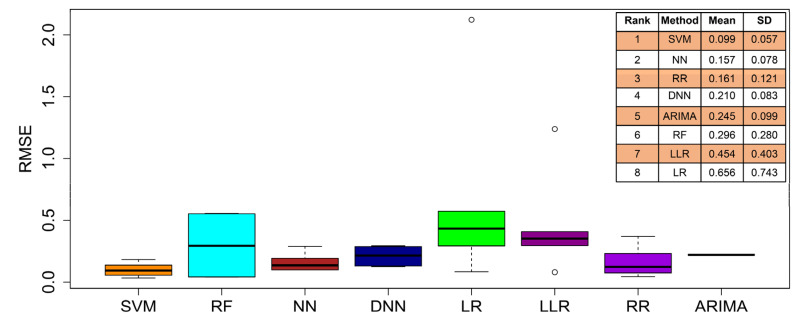
The comparisons of eight methods in the prediction of box-office markets over the years of 2011 to 2016. The box-plots refer to the RMSE values of the predictions listed in Table 1, in which ‘ARIMA’ plots are only the mean value for the different predictor variables with the other methods. In the vertical table, these methods are ranked according to their means of RMSE. ‘SD’ refers to the standard deviation of RMSE.

In these empirical experiments, we noticed that different methods achieved different RMSE values with different predictor variables. In order to obtain the entire rank used to compare all these prediction methods, we performed a statistics summary on the RMSEs. Figure 3 demonstrates the box-plots of RMSE values in these tests. The ranks of individual methods are also shown in the vertical table in Figure 3. We find that SVM-based predictions achieve the lowest RMSE mean and standard deviation (SD). The results directly confirm the use of the SVM method as the best to perform the predictions of box-office revenues from an integrative perspective.

### 3.2. Selection of Prediction Variables

The dependent variables in the prediction methods are GDP and/or NMS. GDP is related to the federal income of economy. It is a significant signature of the running property of the whole country and indicates its development status. NMS is obviously related to the local aspect of the performance of movies. We tested these predictor variables individually and in combination with each method. Compared to the GDP of a country, NMS directly refers to the resources of the movie industry, which enable the interactions of the customers with movies.

The other economic factors, such as per capita income, number of released movies and admissions/tickets sold, also seem to be related to the cultural consumption of box-offices, although they are often difficult to summarize and to access in terms of movie demographics. Moreover, our prediction of nationwide box-offices for one year is based on the economic factors of that year. For practical forecasting, the values of predictor variables are also not available ahead of time. Thus, the early prediction of these economic factors should be a necessity [30,31]. Fortunately, some organizations provide considerable predictions for GDP at the beginning of each year. Utilizing the availability of GDP for the future year obtained from third-party statistical bureaus, we can focus on the prediction of box-office markets with GDP as the selected predictor variable using the SVM-based method.

In the prediction performances, we achieved lower RMSEs in the prediction of the box-office markets in the US than those in China, e.g., 0.056 versus 0.183 by SVM using GDP. In ARIMA, the autoregression is only based on the history of box-office markets. This inspired us to use box-office data of previous years as the dependent variables in the predictions. For validation purposes, we conducted the predictions using the alternative predictors of box-offices in the previous five years. Table 2 illustrates the prediction performances through various feature coding schemes with more predictor variables for the box-office. We find that prediction is not effective (RMSE of 0.494) when we only use the box-office markets as the predictor variables for the SVM method. More experiments show that we can slightly improve the predictions by combining BOX with GDP and/or NMS, e.g., 0.114 using ‘NMS + BOX’ versus 0.121 using only ‘NMS’. In our prediction strategy, the alternative dependent variables are very flexible and can be integrated when they are available. If the statistics of the other economic factors are available, we can easily integrate them as predictor variables. From the perspectives of early availability and convenience feasibility, GDP is a suitable variable for box-office prediction.

### 3.3. Predictions for the US and China Markets

We implement our predictions by various machine learning methods with diverse predictors. From previous comparative studies, we find that there is no prediction methods which consistently achieve the best performance with the lowest RMSE in both countries. However, the SVM-based method with GDP performs relatively and consistently better. Importantly, the predictor variables are easily available if employing the economic GDP predictions. Thus, we focus on our predictions using the SVM-based method with GDP.

**Figure 4:**
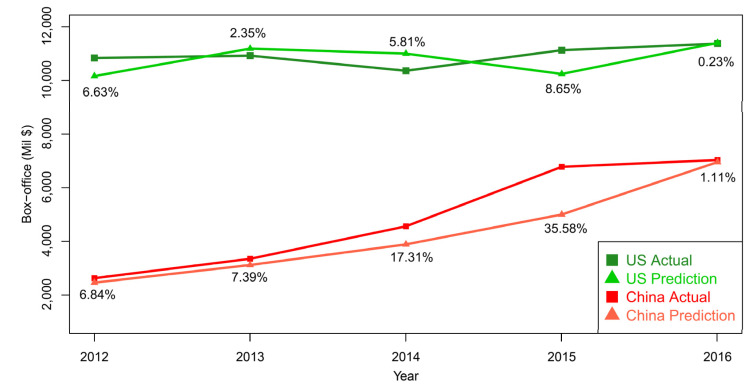
The predicted performance of the box-office markets in US and China over the period of 2012 to 2016 using SVM as the prediction method with GDP as the prediction variable. The number refers to the prediction error (RAPE × 100%) of each year.

Figure 4 demonstrates the prediction and actual curves in the US and China from 2012 to 2016. The prediction RAPE of each year is also shown simultaneously. In 2015, we find the predictions in the two countries have relatively higher errors (8.65% in the US and 35.58% in China). These results indicate turbulences, such as the intellectual property of movie markets in the US and China in 2015. They might contain special movie releases or other anomaly conditions in the movie marketing. The tendency towards high RAPEs seems to start in 2014. In the two time points, the rationality of correspondence between the response variable (box-office) and the dependent variables (GDP) might be interrupted by these turbulences. In the other years, the predictions were close to the actual performance with low errors.

### 3.4. Predictions in 2017

**Figure 5:**
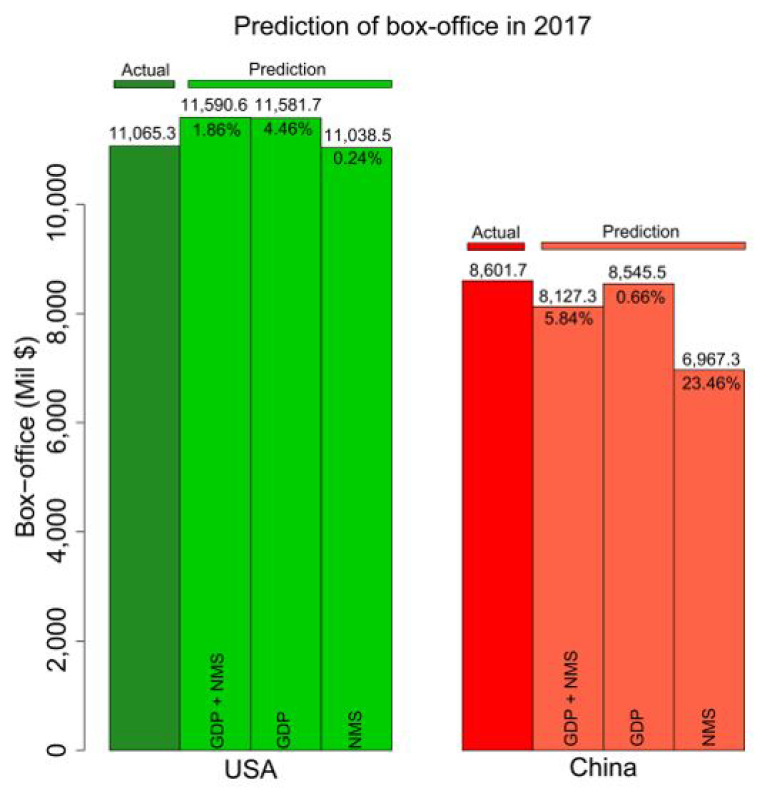
The prediction and actual performance of the box-office markets of 2017 in the US and China. We implemented our predictions using SVM with different predictors, i.e., ‘GDP + NMS’, ‘GDP’ and ‘NMS’. Integrative or individually, the GDPs and NMS in the last five years (2012–2016) were implemented to predict the box-office markets in 2017.

At the beginning of year 2018, the box-office, GDP and NMS of 2017 were announced. In order to further check the prediction accuracy of our proposed method, we validated the predictions and the actual box-office performance of 2017. Figure 5 illustrates the comparison between predictions and actual data. We find the lowest prediction RAPEs are 0.024 and 0.066, using different dependent variables in US and China, respectively. The combination of GDP and NMS reached the lowest prediction error (1.86%) in the US. In China, only the GDP predictor achieved the best prediction with the lowest error (0.66%). The independent validations of predictions in 2017 provide more evidence for the effectiveness and efficiency of our proposed method. 

In our predictions, we implemented the predictions using GDP and/or NMS. Due to the lack of availability of time-series data, the availability of NMS is more difficult than GDP. At the beginning of a new year, the IMF or national statistics bureaus will often release the real-time predictions of the GDP for individual countries for the coming year, while the NMS of the predicted year is not available. Thus, it is more convenient to implement the prediction by using only the prospective GDP as the predictor variable. For instance, the IMF projected the economic growth of the US and China’s GDP to be 2.7% and 6.6%, respectively, in 2018. Integrating the estimated GDPs as well as using their past data, we were able to predict the box-office markets of the two countries in 2018 at the beginning of that year. From an early prediction perspective, our method is very flexible when these economic predictions are available. See the next subsection for more details on this issue.

### 3.5. Consecutive Predictions in 2018 and 2019

In previous comparison studies, GDP was identified as playing a critical role in our prediction strategy. Although it is challenging to predict GDP growth, there are some projections using computational and statistical methods [30]. Fortunately, the projected GDP of the US and China for 2018 and 2019 were released by international or national agencies ahead of the respective year. In fact, the IMF releases these early predictions of GDP prior to each new year. By using the projected GDP, we implemented our prediction method based on the SVM to briefly predict the box-office markets in the two countries in 2018 and 2019 at the beginning of 2018. That is to say, the prediction was able to be implemented two years ahead, as soon as the projected GDP became available. It is practical and flexible in real conditions.

Table 3 illustrates these predicted movie box-offices in 2018 and 2019. For validation, we have also listed the real box-office revenue and the real GDP. For the movie box-offices of the US and China, our proposed method achieves a RAPEs of 6.24% and 5.43% in 2018, and 1.96% and 1.46% in 2019, respectively. Compared with the values in 2018, the projected GDP of both countries was relatively close to its actual value in 2019. Thus, the predictions of movie box-offices achieved a better performance in 2019. The mean RAPEs in the two consecutive years’ predictions hit 4.1% and 3.445% for the US and China, respectively. The results prove the effectiveness and efficiency of our method in predicting movie box-office markets using the economic factor of GDP. Additionally, the results indicate the strong association between the economic factor and box-office market. If we obtain a precise projection of the GDP for one country from the IMF ahead of every year, our proposed method is expected to provide an accurate prediction of movie box-office revenue for the corresponding country.

The gross box-office market prediction ahead of every year can provide valuable directions for potential developments in the movie industry. Due to the dynamics of economics, governmental organizations, such as the IMF, often adjust their projected GDPs according to the conditions of economic situations. Obviously, our predictions can also be adjusted accordingly when the new projected GDP is updated. Similarly, when the real-time number of NMS are released for these coming years, we can also employ the projected numbers to obtain new predictions. These predictions over several consecutive years prove the flexibility of our proposed prediction strategy.

## 4. Conclusions

In this work, we provided a machine learning-based method for predicting the nationwide box-office markets of movie revenue using economic factors. We compared the prediction performances of eight methods to identify the superiority of the SVM-based method for our forecasting. We also evaluated the predictions using different combinations of dependent variables of economic factors, i.e., GDP, the available resources in movie industry, i.e., NMS, as well as the past data of box-offices, i.e., BOX. Given the easy availability of GDP statistics and estimations, it was identified as the most applicable prediction variable in the SVM-based prediction method. The precise predictions provide direct evidence for the effectiveness and efficiency of our proposed strategy. The comparison studies of different prediction methods with different combinations of the dependent factors in the time-series studies of the box-office indicate the advantages of our developed method.

Moreover, our proposed method is general and is flexible enough to be extended. It can be easily applied to predict other important incomes in cultural industries, such as books, music and copyright. It is also expected to include more intricate indices or attributors for describing complex phenomena [32,33] in cultural production and consumption. The alternative artificial intelligence strategy of handling the uncertainty of complex economic systems is also a direction our research will take in the future [34]. These predictions will deeply benefit the future and potential understanding of the tendencies of economic development in the industry, especially when the time-series data of days, weeks, months and quarters are available. The crucial patterns of the time-series movie revenues will greatly benefit related investment and other decision making.

## Figures and Tables

**Table 1:** The prediction performances of box-offices in the US and China by eight methods using GDP and/or NMS.

Method	Predictor Variable	Country	RAPE					RMSE
			2012	2013	2014	2015	2016	
SVM	GDP	US	0.066	0.024	0.058	0.086	0.002	0.056
		China	0.068	0.074	0.173	0.356	0.011	0.183
	NMS	US	0.052	0.026	0.026	0.037	0.002	**0.033**
		China	0.058	0.075	0.008	0.117	0.225	**0.121**
	GDP + NMS	US	0.092	0.041	0.059	0.083	0.019	0.065
		China	0.063	0.069	0.085	0.175	0.219	0.137
RF	GDP	US	0.042	0.035	0.035	0.048	0.043	0.041
		China	0.537	0.509	0.586	0.761	0.235	**0.552**
	NMS	US	0.039	0.030	0.043	0.045	0.045	0.041
		China	0.532	0.529	0.599	0.718	0.234	0.546
	GDP + NMS	US	0.04	0.034	0.035	0.044	0.046	**0.040**
		China	0.539	0.509	0.613	0.753	0.226	0.555
NN	GDP	US	0.084	0.092	0.036	0.113	0.138	0.098
		China	0.021	0.079	0.252	0.554	0.199	0.289
	NMS	US	0.078	0.087	0.031	0.107	0.132	**0.093**
		China	0.262	0.228	0.081	0.046	0.239	0.193
	GDP + NMS	US	0.079	0.088	0.032	0.108	0.133	0.094
		China	0.153	0.018	0.034	0.321	0.139	**0.172**
DNN	GDP	US	0.104	0.141	0.043	0.182	0.140	0.131
		China	0.415	0.213	0.064	0.361	0.243	0.287
	NMS	US	0.089	0.110	0.135	0.153	0.130	**0.125**
		China	0.403	0.274	0.089	0.351	0.240	0.292
	GDP + NMS	US	0.129	0.159	0.061	0.136	0.208	0.147
		China	0.491	0.271	0.062	0.196	0.198	**0.281**
LR	GDP	US	0.490	1.176	0.099	0.111	0.038	0.574
		China	0.292	0.398	0.105	0.369	0.189	**0.292**
	NMS	US	0.174	0.041	0.034	0.024	0.028	**0.083**
		China	4.617	0.497	0.953	0.039	0.220	2.122
	GDP + NMS	US	0.49	1.176	0.099	0.111	0.038	0.574
		China	0.292	0.398	0.105	0.369	0.189	0.292
LLR	GDP	US	0.607	0.662	0.100	0.108	0.040	0.408
		China	0.221	0.519	0.133	0.232	0.215	**0.295**
	NMS	US	0.166	0.045	0.034	0.023	0.027	**0.080**
		China	2.433	0.838	0.998	0.171	0.158	1.239
	GDP + NMS	US	0.607	0.662	0.100	0.108	0.040	0.408
		China	0.221	0.519	0.133	0.232	0.215	**0.295**
RR	GDP	US	0.176	0.054	0.02	0.113	0.049	0.100
		China	0.181	0.047	0.254	0.405	0.047	0.231
	NMS	US	0.057	0.004	0.067	0.041	0.017	**0.044**
		China	0.586	0.507	0.111	0.018	0.268	0.370
	GDP + NMS	US	0.104	0.050	0.054	0.090	0.055	0.074
		China	0.086	0.134	0.016	0.063	0.281	**0.147**
ARIMA	BOX	US	0.665	0.509	0.382	0.287	0.176	**0.438**
		China	0.063	0.035	0.120	0.175	0.252	**0.151**

**Table 2:** Comparisons of prediction performance using the SVM-based method with alternative predictor variables to ‘BOX’, in China.

Predictor Variable	RAPE					RMSE
	2012	2013	2014	2015	2016	
BOX	0.577	0.361	0.495	0.740	0.326	0.494
GDP + BOX	0.044	0.015	0.093	0.187	0.149	0.117
NOS + BOX	0.056	0.045	0.027	0.153	0.190	0.114
GDP + NOS + BOX	0.021	0.010	0.086	0.255	0.029	0.121

**Table 3:** The prediction of box-office markets in the US and China using the estimated GDPs in 2018 and 2019.

Country	Variable	2018			2019		
		Predicted	Actual	RAPE	Predicted	Actual	RAPE
US	Box office (Mil $)	11,147.17	11,889.3	6.24%	11,542.76	11,320.9	1.96%
	Projected GDP (Bil $)	20,351.8	20,580.2	1.11%	21,239.30	21,433.2	0.91%
China	Box office (Mil $)	9890.37	9380.92	5.43%	10,030.98	9887.08	1.46%
	Projected GDP (Bil $)	13,552.08	14,142.78	4.18%	14,432.96	15,244.08	5.32%

## Data Availability

All the data are available from public domains. They are available upon request from the corresponding author.
